# SBF-1 exerts strong anticervical cancer effect through inducing endoplasmic reticulum stress-associated cell death via targeting sarco/endoplasmic reticulum Ca^2+^-ATPase 2

**DOI:** 10.1038/cddis.2014.538

**Published:** 2014-12-18

**Authors:** W Li, Z Ouyang, Q Zhang, L Wang, Y Shen, X Wu, Y Gu, Y Shu, B Yu, X Wu, Y Sun, Q Xu

**Affiliations:** 1 State Key Laboratory of Pharmaceutical Biotechnology, School of Life Sciences, Nanjing University, 22 Hankou Road, Nanjing 210093, China; 2 Department of Oncology, The First Affiliated Hospital of Nanjing Medical University, Nanjing 210029, China; 3 State Key Laboratory of Bio-organic and Natural Products Chemistry, Shanghai Institute of Organic Chemistry, Chinese Academy of Sciences, Shanghai 200032, China

## Abstract

Cervical cancer is one of the most common carcinomas in the genital system. In the present study, we report that SBF-1, a synthetic steroidal glycoside, has a strong antigrowth activity against human cervical cancer cells *in vitro* and *in vivo*. SBF-1 suppressed the growth, migration and colony formation of HeLa cells. In addition, severe endoplasmic reticulum (ER) stress was triggered by SBF-1, and 4-phenyl-butyric acid, a chemical chaperone, partially reversed SBF-1-induced cell death. To uncover the target protein of SBF-1, the compound was labeled with biotin. The biotin-labeled SBF-1 bound to sarco/ER Ca^2+^-ATPase 2 (SERCA2) and colocalized with SERCA2 in HeLa cells. Moreover, SBF-1 inhibited SERCA activity, depleted ER Ca^2+^ and increased cytosolic Ca^2+^ levels. 1,2-Bis(*o*-aminophenoxy)ethane-*N*,*N*,*N*′,*N*′-tetraacetic acid, a chelator of Ca^2+^, partially blocked SBF-1-induced ER stress and growth inhibition. Importantly, knockdown of SERCA2 increased the sensitivity of HeLa cells to SBF-1-induced ER stress and cell death, whereas overexpression of SERCA2 decreased this sensitivity. Furthermore, SBF-1 induced growth suppression and apoptosis in HeLa xenografts, which is closely related to the induction of ER stress and inhibition of SERCA activity. Finally, SERCA2 expression was elevated in human cervical cancer tissues (*n*=299) and lymph node metastasis (*n*=8), as compared with normal cervix tissues (*n*=23), with a positive correlation with clinical stages. In all, these results suggest that SBF-1 disrupts Ca^2+^ homeostasis and causes ER stress-associated cell death through directly binding to SERCA2 and inhibiting SERCA activity. Our findings also indicate that SERCA2 is a potential therapeutic target for human cervical cancer.

The endoplasmic reticulum (ER) is a membranous system that is essential for the function and survival of mammalian cells.^1^ To achieve an optimum state for survival, a cell may undergo various pathways in the ER, such as ones that regulate protein folding, posttranslational modifications, lipid and steroid synthesis, gene expression, cellular metabolism and calcium signaling. When the pathways are disturbed, ER functions become overwhelmed and the accumulation of misfolded proteins within the ER lumen ultimately leads to ER stress and initiates the unfolded protein response (UPR) to restore ER proteostasis.^2, 3^ The UPR is generally considered to be the transcriptional induction of molecular chaperones in response to ER stress.^4^ Three ER stress arms have been identified so far: protein kinase RNA-like ER kinase (PERK), inositol-requiring enzyme-1 (IRE1) and activating transcription factor-6 (ATF6).^3, 5, 6^ These three ER stress arms can act as coregulators of most targets to ensure the redundancy and robustness of this adaptive response.^3, 7^ If the response fails or prolongs, apoptotic cell death ensues.^3, 8^

Calcium homeostasis is involved in a multitude of signaling in ER, in which calcium is actively accumulated by sarco/ER Ca^2+^-ATPase (SERCA) transport ATPases.^9, 10^ As SERCA-dependent calcium transport is the only calcium uptake mechanism in this organelle, the regulation of SERCA function by the cell constitutes a key mechanism to adjust calcium homeostasis in the ER depending on the cell type and its state of differentiation.^10^ The SERCA pump is encoded by a family of three genes, *SERCA1*, *2* and *3*, which are highly conserved but localized on different chromosomes.^11^ At present, more than 10 different SERCA isoforms have been discovered in this family. These isoforms exhibit both tissue and developmental specificity, suggesting that they contribute to unique physiological properties of the tissue in which they are expressed.^11, 12^ SERCA expression levels can undergo significant changes during cell differentiation or tumorigenesis, leading to modified ER calcium storage.^10^ For example, expression levels of SERCA3, a lower calcium affinity calcium pump, are highly variable. In several cell systems, SERCA3 expression is selectively induced during differentiation, whereas during tumorigenesis SERCA3 expression is decreased.^10, 13, 14, 15, 16, 17, 18^ Diverse levels of SERCA2 are also correlated with tumorigenesis.^19, 20, 21^

Cervical cancer is the third most common carcinoma in women worldwide.^22, 23, 24^ The American Cancer Society estimates that 12 360 new cases and 4020 deaths of cervical cancer are projected to occur in the United States in 2014.^22^ Despite more and more efforts are being made to improve therapy, a significant proportion of women still die from recurrence and chemoresistance,^24^ which is a hallmark of cancer cells.^25, 26^ Searching for an efficient agent is still a requirement for the therapy of the cancer. Among such efforts, induction of ER stress by targeting SERCA might be a potential therapeutic strategy to treat apoptosis-resistant cancer cells.^27, 28^ Our previous study identified that SBF-1, a synthetic steroidal glycoside, had a very strong antitumor activity in various cancer types.^29, 30^ Herein, we report a new property of SBF-1 for characterizing its anticancer activity as a SERCA inhibitor that directly binds to SERCA2. In human cervical cancer cells, ER Ca^2+^ homeostasis is disrupted and ER stress-mediated cell death is induced by SBF-1 both *in vitro* and *in vivo*.

## Results

### SBF-1 induces ER stress-associated cell death in human cervical cancer HeLa cells

**Figure 1:**
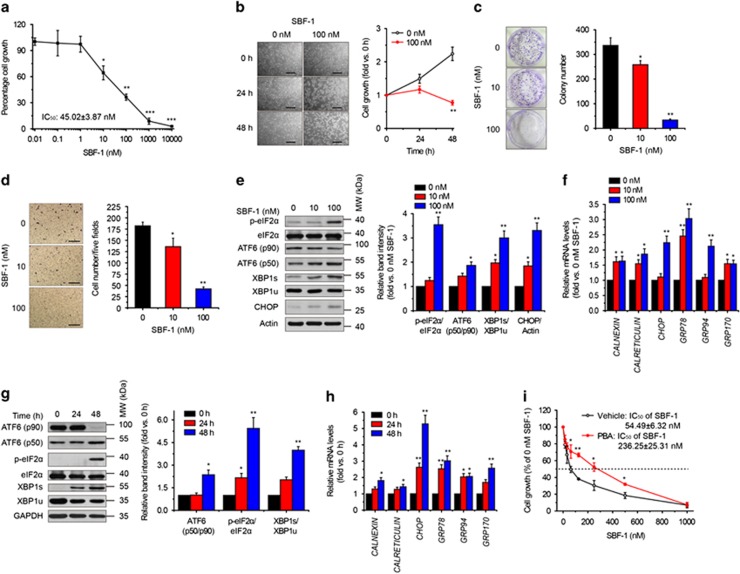
SBF-1 induced ER-stress-associated cell death in human cervical cancer HeLa cells. (**a** and **b**) HeLa cells (2 × 10^3^ per well) were seeded into 96-well plates and incubated with DMSO (0.1%) or indicated concentrations of SBF-1 for indicated time periods. Cell growth was determined by MTT assay. (**c** and **d**) HeLa cells (2 × 10^5^ per well) were seeded into six-well plates and incubated with DMSO (0.1%) or various concentrations of SBF-1 for 48 h. (**c**) One thousand viable cells per well were seeded into six-well plates and maintained at 37 °C for another 2 weeks. Colonies were fixed with 4% PFA and stained with crystal violet. The number of colonies (>80 cells per colony) was counted under a light microsope. (**d**) Ten thousand cells (in 0.1 ml serum-free DMEM) were seeded into the inner room of transwells and maintained at 37 °C for another 24 h. Migrated cells were fixed in 4% PFA and stained with crystal violet. The number of migrated cells was calculated under a light microscope. (**e** and **f**) HeLa cells (2 × 10^5^ per well) were seeded into six-well plates and incubated with DMSO (0.1%) or various concentrations of SBF-1 for 48 h. (**e**) The protein levels of ATF6*α* (p90), ATF6*α* (p50), phosphorylated eIF2*α*^Ser51^, XBP1u, XBP1s and CHOP were detected by immunoblotting (left panel), and the relative band intensity was analyzed with Image J software (NIH, right panel) and normalized with *β*-actin. *β*-Actin was performed as a loading control. (**f**) The mRNA levels of *CALNEXIN*, *CALRETICULIN*, *CHOP*, *GRP78*, *GRP94* and *GRP170* were detected by quantitative real-time PCR (Q-PCR) and normalized with *RN18S*. (**g** and **h**) HeLa cells (2 × 10^5^ per well) were seeded into six-well plates and incubated with DMSO (0.1%) or 100 nM SBF-1 for indicated time periods. (**g**) The protein levels of ATF6*α* (p90), ATF6*α* (p50), phosphorylated eIF2*α*^Ser51^, XBP1u and XBP1s were detected by immunoblotting (left panel), and the relative band intensity were analyzed with Image J software (right panel). Glycolytic glyceraldehyde-3-phosphate dehydrogenase (GAPDH) was performed as a loading control. (**h**) The mRNA levels of *CALNEXIN*, *CALRETICULIN*, *CHOP*, *GRP78*, *GRP94* and *GRP170* were detected by Q-PCR and normalized with *RN18S*. (**i**) HeLa cells (2 × 10^3^ per well) were seeded into 96-well plates and incubated with vehicle (0.2% DMSO) or PBA (2.5 mM) plus various concentrations of SBF-1 for 48 h. Then, cell growth was determined by MTT assay. The dotted line showed 50% cell growth of 0 nM SBF-1. Images shown were representative of three independent experiments. Scale bars, 100 *μ*m. Data were means±S.D. of three independent experiments. *P<0.05, **P<0.01 *versus* 0 nM SBF-1 (**a** and **c**–**f**), 0 h (**b**, **g** and **h**) or vehicle (**i**). MW, molecular weight

As shown in Figure 1, SBF-1 concentration- (Figure 1a) and time-dependently (Figure 1b) inhibited growth of a human cervical cancer cell line (HeLa). Colony formation (Figure 1c) and migration ability (Figure 1d) of HeLa cells were also significantly repressed by SBF-1 treatment. Then, we observed that protein levels of ATF6 (N terminal, p50), spliced form of X-box-binding protein 1 (XBP1s), phospho-eukaryotic translation initiation factor 2A (eIF2*α*^Ser51^) and C/EBP homologous protein (CHOP) were significantly increased after exposure to 100 nM SBF-1 (Figure 1e). The mRNA levels of *CALNEXIN*, *CALRETICULIN*, *CHOP*, glucose-regulated protein 78 (*GRP78*), *GRP94* and *GRP170* were also significantly increased by 100 nM SBF-1 (Figure 1f). Moreover, 100 nM SBF-1 time-dependently increased the protein levels of ATF6 (p50), XBP1s and phospho-eIF2*α*^Ser51^ (Figure 1g), and increased the mRNA levels of *CALNEXIN*, *CALRETICULIN*, *CHOP*, *GRP78*, *GRP94* and *GPR170* (Figure 1h). 4-Phenyl-butyric acid (PBA), a chemical chaperone reported to be an inhibitor of ER stress,^27, 31^ partially reversed SBF-1-induced growth inhibition, where PBA increased the 50% inhibitory concentration (IC_50_) of SBF-1 from 54.49±6.32 to 236.25±25.31 nM (Figure 1i). These results suggest that SBF-1-induced growth inhibition of HeLa cells is associated with ER stress.

### SBF-1 binds to SERCA2 and increases the intracellular Ca^2+^ levels

**Figure 2:**
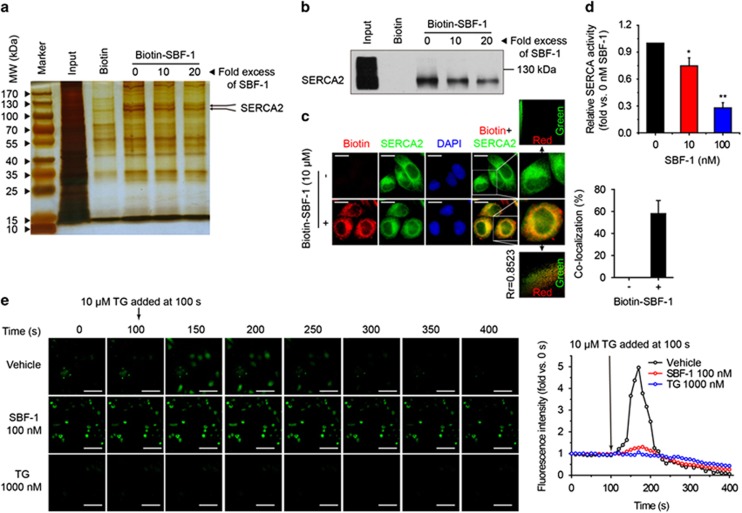
SBF-1 bound to SERCA2 and caused depletion of ER Ca^2+^ in HeLa cells. (**a** and **b**) HeLa whole-cell lysates were incubated with biotin (10 *μ*M), biotin-SBF-1 (10 *μ*M), biotin-SBF-1 (10 *μ*M) plus SBF-1 (100 *μ*M) or biotin-SBF-1 (10 *μ*M) plus SBF-1 (200 *μ*M), and streptavidin-conjugated sepharose beads. (**a**) The proteins bound to the beads were separated with SDS-PAGE and stained with silver nitrate. The arrows indicated the interested protein bands analyzed by LC/MS. (**b**) The proteins bound to the beads were separated with SDS-PAGE and immunoblotted with anti-SERCA2 antibody. (**c**) HeLa cells were seeded on glass slides and incubated with DMSO (0.1%) or biotin-SBF-1 (10 *μ*M) for 10 min. Then, the cells were fixed in 4% PFA and stained with anti-biotin and anti-SERCA2 antibodies. Red, green and blue color indicated the location of biotin, SERCA2 and nucleus, respectively. The yellow color indicated the colocalization of biotin-SBF-1 and SERCA2. Scale bars, 5 *μ*m. The colocalization rate was analyzed with Image Pro Plus 6.0 software. (**d**) HeLa cells pretreated with DMSO (0.1%) or various concentrations of SBF-1 for 48 h were washed once with cold saline (0.9% NaCl) and lysed with pulse sonication. Then, SERCA activity was measured and normalized with DMSO-treated cells (SERCA activity of DMSO-treated cells was 1.66±0.22 *μ*mol/mg protein per h). *P<0.05, **P<0.01 *versus* 0 nM SBF-1. (**e**) HeLa cells pretreated with vehicle (0.1% DMSO), SBF-1 (100 nM) or thapsigargin (TG, 1 *μ*M) were stained with Fluo-4 AM (2.5 *μ*M). Fluorescence signals were acquired under a mercury lamp (Olympus). Photos were taken every 10 s and 10 *μ*M TG was added at 100 s when the signals were stable. Fluorescence intensity was analyzed with Image Pro Plus 6.0 software (Media Cybernetics) and normalized with the intensity of 0 s. Scale bars, 40 *μ*m. Images shown were representative of three independent experiments

**Figure 3:**
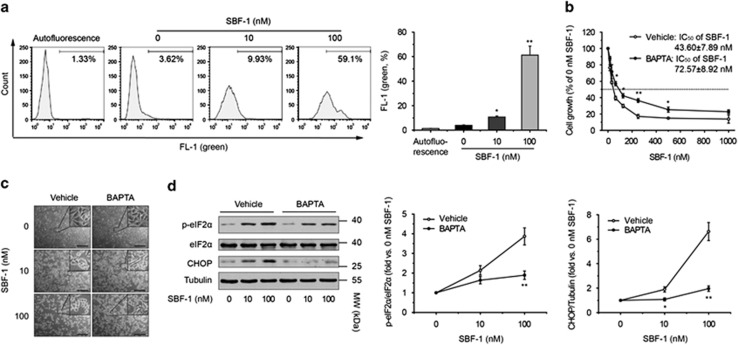
ER stress induced by SBF-1 was partially dependent on the increase of cytosolic Ca^2+^. (**a**) HeLa cells pretreated with DMSO (0.1%) or various concentrations of SBF-1 for 48 h. Then, the cells were stained with Fluo-4 AM (2.5 *μ*M). Percentage of Fluo-4-positive cells was analyzed with FACS. (**b**) HeLa cells (2 × 10^3^ per well) were seeded into 96-well plates and incubated with vehicle (0.1% DMSO) or 10 *μ*M BAPTA-am for 1 h. Then, the cells were incubated with DMSO (0.1%) or various concentrations of SBF-1 for another 48 h. Cell growth was determined by MTT assay. The dotted line indicated 50% cell growth of 0 nM SBF-1. (**c** and **d**) HeLa cells were seeded into six-well plates and incubated with vehicle (0.1% DMSO) or BAPTA-am (10 *μ*M) for 1 h. Then, the cells were incubated with DMSO (0.1%) or various concentrations of SBF-1 for 48 h. (**c**) Photos produced by phase contrast microscope were shown. (**d**) The protein levels of phosphorylated eIF2*α*^Ser51^, total eIF2*α* and CHOP were detected by immunoblotting (left panel), and the relative band intensity was analyzed with Image J software and normalized with *α*-tubulin (middle and right panels). *α*-Tubulin was performed as a loading control. Images shown were representative of more than three independent experiments. Data were means±S.D. of more than three independent experiments. Scale bars, 100 *μ*m. *P<0.05, **P<0.01 *versus* 0 nM SBF-1 (**a**) or vehicle (**b**–**d**)

To find out binding proteins of SBF-1, SBF-1 was labeled with biotin (Supplementary Figure S2a). The biotin conjugate of SBF-1 (biotin-SBF-1) still showed a strong antigrowth activity (IC_50_, 436.63±48.79 nM) despite an obvious decrease as compared with SBF-1 (IC_50_, 45.66±6.27 nM; Supplementary Figure S2b). Biotin-SBF-1 was then incubated with HeLa whole-cell lysates and streptavidin-conjugated sepharose beads in the presence or absence of 10- to 20-fold excess of SBF-1. The proteins bound to the beads were separated with SDS-PAGE and the bands between 100 and 130 kDa were cut and analyzed with liquid chromatography-mass spectrometry (LC/MS). Sarco/ER Ca^2+^-ATPase 2 (SERCA2), the most abundant SERCA isoform in HeLa cells (Supplementary Figure S3a), was identified to be a binding protein of SBF-1 (Figures 2a and b), and biotin-SBF-1 colocalized with SERCA2 in HeLa cells (Figure 2c). Furthermore, SERCA activity of HeLa cells was significantly suppressed by both 10 and 100 nM SBF-1 (Figure 2d) and the protein level of SERCA2 was compensatorily increased (Supplementary Figures S3b and c), whereas the mRNA level of *SERCA2* was not changed (Supplementary Figure S3d). Moreover, ER Ca^2+^ was depleted (Figure 2e) and intracellular Ca^2+^ levels were significantly increased by exposure to 100 nM SBF-1 in both a concentration- and time-dependent manner (Figure 3a and Supplementary Figure S4). BAPTA (1,2-Bis(o-aminophenoxy)ethane-*N*,*N*,*N*′,*N*′-tetraacetic acid), a cell-permeant Ca^2+^ chelator widely used in Ca^2+^-related study,^27, 32^ partially reversed SBF-1-induced proliferation suppression (Figure 3b) and markedly attenuated ER stress induced by SBF-1 (Figures 3c and d) in HeLa cells.

### The SERCA2 level controls the sensitivity of HeLa cells to SBF-1

**Figure 4:**
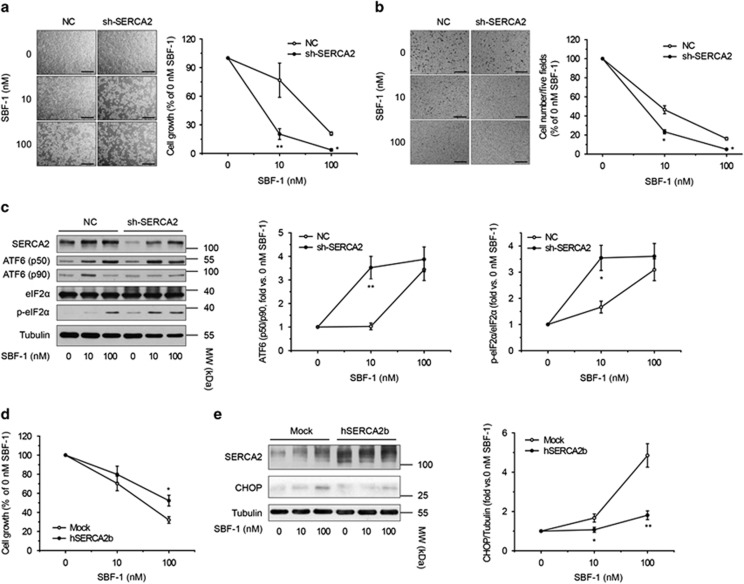
SERCA2 level controlled the sensitivity of HeLa cells to SBF-1. (**a**–**c**) HeLa cells stably infected with NC shRNA and SERCA2 shRNA were incubated with DMSO (0.1%) or various concentrations of SBF-1 for 48 h. (**a**) Representative images of cell growth of three independent experiments were shown. Scale bars, 100 *μ*m. Cell number was counted by Typan blue staining and normalized with 0 nM SBF-1. (**b**) Ten thousand viable cells (in 0.1 ml serum-free DMEM) were seeded into the inner rooms of transwells and maintained at 37 °C for another 24 h. Migrated cells were fixed, stained with crystal violet and counted under a light microscope. Representative images of three independent experiments were shown (left panel). Scale bars, 100 *μ*m. The number of migrated cells was counted with Image J software (right panel). (**c**) The protein levels of SERCA2, ATF6*α* (p90), ATF6*α* (p50), total eIF2*α* and phosphorylated eIF2*α* were detected by immunoblotting (left panel), and the relative band intensity was analyzed with Image J software and normalized with *α*-tubulin (middle and right panels). *α*-Tubulin was performed as a loading control. (**d** and **e**) HeLa cells transiently transfected with mock or hSERCA2b for 48 h were incubated with various concentrations of SBF-1 for 48 h. (**d**) Cell growth was determined by Trypan blue staining and normalized with DMSO-treated cells. (**e**) The protein levels of SERCA2 and CHOP were detected by immunoblotting (left panel), and the relative band intensity was analyzed with Image J software and normalized with *α*-tubulin (right panel). *α*-Tubulin was performed as a loading control. Images shown were representative of three independent experiments. Data were means±S.D. of three independent experiments. *P<0.05, **P<0.01 *versus* NC (**a**–**c**) or mock (**d** and **e**). NC, negative control

To further demonstrate the role of SERCA2 in the antitumor effects of SBF-1, we stably knocked down SERCA2 in HeLa cells (Supplementary Figures S5a and b), which had no influences on the growth of HeLa cells under normal culture conditions (Supplementary Figure S5c). However, the SERCA2 silence greatly enhanced SBF-1-induced repression of cell growth (Figure 4a) and migration (Figure 4b). Then, we confirmed that the protein levels of ATF6*α* (p50) and phospho-eIF2*α*^Ser51^ (Figure 4c), and the mRNA levels of *CHOP* and *GRP78* (Supplementary Figure S6) in HeLa cells with stable SRECA2 knockdown were increased more significantly after exposure to SBF-1, compared with cells with stable NC lentivirus infection. In addition, SERCA2b overexpression had no influences on the growth of HeLa cells under normal culture conditions (Supplementary Figure S7), but partially reduced SBF-1-induced proliferation suppression (Figure 4d). The increase in protein levels of CHOP by SBF-1 was almost completely blocked in HeLa cells transfected with hSERCA2b as compared with cells transfected with mock vector (Figure 4e). The above results indicate that SBF-1 suppresses the HeLa cell growth and migration depending on the activity and level of SERCA2.

### SBF-1 inhibits tumor growth at a very low dose in HeLa xenografts with decreased SERCA activity and increased ER stress and apoptosis

**Figure 5:**
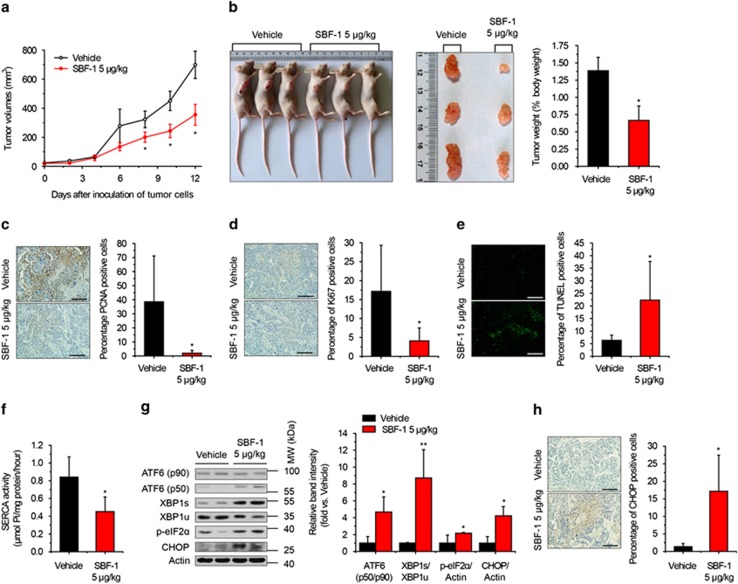
SBF-1 suppressed tumor growth of HeLa xenografts in nude mice. One million cells (in 0.1 ml PBS) were injected subcutaneously into the right flanks of female nude mice (6–8 weeks old). One week after the inoculation, all the mice formed visible tumors and were distributed into two groups. Vehicle (0.1 DMSO in PBS, *n*=5) and 5 *μ*g/kg SBF-1 (*n*=6) were intraperitoneally injected into the mice. (**a**) Tumor volumes of the mice. (**b**) Three representative mice per group were shown (left panel). Three representative tumors were shown (middle panel), and the percentage of tumor weight in body weight was calculated (right panel). (**c**) The protein level of PCNA was detected with immunohistochemistry. Representative images of six mice were shown (left panel), and the percentage of PCNA-positive cells was calculated (right panel). (**d**) The protein level of Ki-67 was detected by immunohistochemistry. Representative images of six mice were shown (left panel), and the percentage of Ki-67-positive cells was calculated (right panel). (**e**) Apoptosis in tumor sections was detected with TUNEL staining. Representative images of six mice were shown (left panel), and the percentage of TUNEL-positive cells was calculated (right panel). (**f**) SERCA activity of vehicle- and SBF-1-treated xenografts. (**g**) The protein levels of ATF6*α* (p90), ATF6*α* (p50), XBP1s, XBP1u, phosphorylated eIF2*α* and CHOP were detected by immunoblotting (left panel), and the relative band intensity was analyzed with Image J software and normalized with *β*-actin (right panel). Blots shown were representative of six mice. (**h**) The protein level of CHOP was detected by immunohistochemistry. Representative images of six mice were shown (left panel), and the percentage of CHOP-positive cells was calculated (right panel). Scale bars, 50 *μ*m. Data were means±S.D. of six mice. *P<0.05, **P<0.01 *versus* vehicle

To evaluate the antitumor effects of SBF-1 *in vivo*, we daily intraperitoneally injected vehicle (0.1% dimethyl sulfoxide (DMSO) in phosphate-buffered saline (PBS)) or 5 *μ*g/kg SBF-1 to tumor-bearing nude mice. Changes in tumor volumes and body weight were measured and recorded every 2 days. The growth of HeLa xenografts was significantly inhibited after 8 days of administration of SBF-1, in comparison with vehicle-treated group (Figure 5a). The tumor mass and the percentage of tumor weight in body weight was significantly decreased by SBF-1 treatment (Figure 5b), whereas body weight was not influenced (Supplementary Figure S8). Furthermore, the expression of proliferating cell nuclear antigen (PCNA) (Figure 5c) and antigen Ki-67 (Figure 5d) was significantly inhibited by SBF-1. In contrast, the percentage of terminal deoxynucleotidyl transferase dUTP nick-end labeling (TUNEL)-positive cells was markedly increased in SBF-1-treated xenografts (Figure 5e). As expected, in this case, the SERCA activity of tumor xenografts was significantly inhibited by SBF-1 (Figure 5f), with increased SERCA2 protein level and unchanged mRNA level (Supplementary Figure S9). Meanwhile, the protein levels of XBP1s, ATF6*α* (p50), phospho-eIF2*α*^Ser51^ and CHOP (Figure 5g), and the percentage of CHOP-positive cells (Figure 5h) were significantly increased in SBF-1-treated xenografts, as compared with vehicle-treated xenografts.

### SERCA2 positively correlates with the malignant progress of human cervical cancer

**Figure 6:**
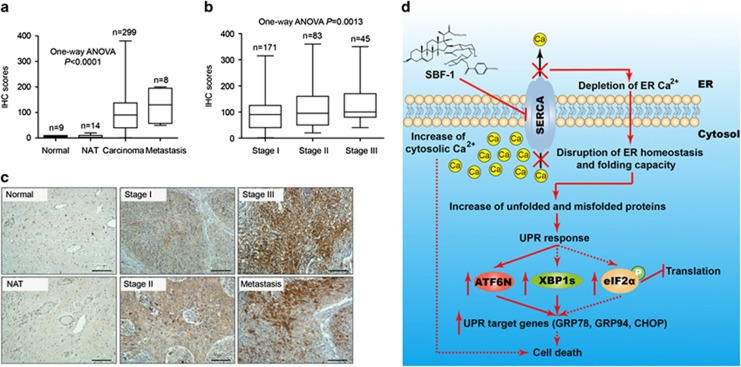
SERCA2 was correlated with the malignance of human cervical cancer. The protein level of SERCA2 in the tissue arrays of normal cervix tissues (normal), cancer adjacent normal cervix tissue (NAT), malignant cervical cancer tissues of different clinical stages (carcinoma, stages I–III) and lymph node metastasis from uterine cervix (metastasis) was detected by immunohistochemistry (IHC). Then, IHC scores were evaluated as shown in Supplementary Figure S10, and the variance was analyzed with one-way ANOVA. (**a**) IHC scores for normal tissues, NAT, carcinoma and metastasis. (**b**) IHC scores for different clinical stages of human cervical cancer. (**c**) Representative images of normal tissues, NAT, different stages of carcinomas and lymph metastasis. Scale bars, 40 *μ*m. (**d**) Illustration for the mechanism underlying the anticervical cancer effects of SBF-1. When cells are exposed to SBF-1, SERCA activity is suppressed, inducing depletion of ER Ca^2+^ and increase of cytosolic Ca^2+^, which disturbs ER folding capacity and increases unfolded and misfolded proteins, activating the signaling pathways of UPR response and causing ER stress-associated cell death. ATF6N, N terminal of ATF6*α*

To evaluate the relationship between SERCA2 and malignance of cervical cancer, the protein level of SERCA2 was detected by immunohistochemistry in tissue arrays of human cervical cancer. Modest SERCA2 expression was detected in normal cervix tissues (*n*=9) and cancer adjacent normal cervix tissue (NAT, *n*=14). Comparably, a significantly elevated SERCA2 expression was detected in malignant tumor tissues (carcinoma, *n*=299) and lymph node metastasis (metastasis, *n*=8) (Figures 6a and c; one-way analysis of variance (ANOVA), *P*<0.0001), and such expression among 299 samples (stage I, *n*=171; stage II, *n*=83; stage III, *n*=45) was increased along with the clinical stages of carcinoma (Figures 6b and c; one-way ANOVA, *P*=0.0013). These results indicate that SERCA2 has a positive correlation with the malignance of human cervical cancer and could be a therapeutic target for cervical cancer therapy.

## Discussion

Cervical cancer is the most common life-threatening disease among women worldwide, and the therapeutic effects are bleak because of recurrence and chemoresistance.^24^ Thus, it is significant to search for effective targets for the treatment of cervical cancer. In the present study, we found that low concentrations of SBF-1 (10 and 100 nM) significantly inhibited the growth, migration and colony formation of HeLa cells, a popularly used human cervical cancer cell line (Figures 1a–d). ER stress is a well-known reason for cell death.^33^ SBF-1 induced ER stress and UPR responses in a concentration- and time-dependent manner, and the chemical chaperone PBA partially reversed the antigrowth activity of SBF-1 (Figures 1e–i). Compared with slowly dividing cells, rapidly dividing cancer cells are more sensitive to ER stress inducers, because of the high levels of protein folding.^2^ Compared with naive T lymphocytes and ECV304 cells (slowly dividing cells), Jurkat and HeLa cells (rapidly dividing cancer cells) were more sensitive to SBF-1 (Figure 1a and Supplementary Figure S11), suggesting that SBF-1 antigrowth effects is not due to general cytotoxicity.

High concentrations of Ca^2+^ in ER lumen are essential for ER folding capacity.^34^ SERCA is a pump to transport Ca^2+^ from the cytoplasm to ER lumen,^11, 12^ and inhibition of SERCA activity impairs ER Ca^2+^ homeostasis and causes severe ER stress.^34, 35, 36, 37^ SERCA2, the major SERCA isoform of HeLa cells, was found to bind with SBF-1, and SERCA activity was suppressed by SBF-1 (Figures 2a–d). Accordingly, SBF-1 depleted ER Ca^2+^ and increased intracellular Ca^2+^ levels, and Ca^2+^ chelator BAPTA partially reversed SBF-1-induced ER stress and growth suppression (Figures 2e and 3). Interestingly, the baseline Ca^2+^ levels in SBF-1-treated cells were obviously higher than those in TG-treated cells (Figure 2e), suggesting the differences between SBF-1 and TG. It is well known that TG is a specific SERCA inhibitor, which depletes ER Ca^2+^ quickly.^27^ After incubation with TG for 48 h, ER Ca^2+^ was almost completely depleted and exhausted. However, there was still abundant cytosolic Ca^2+^, and a small quantity of ER Ca^2+^ in SBF-treated cells. As shown in Figure 2c, the colocalization rate of SBF-1 and SERCA2 was ~60%, indicating that SBF-1 also bound to other proteins, which was supported by the competitive binding assay (Figure 2a). LC/MS identified many other proteins, including plasma membrane calcium-transporting ATPase 1 (PMCA1, data not shown). Moreover, SBF-1 started to increase the baseline cytosolic Ca^2+^ levels after 3-h incubation (Supplementary Figure S4) and did not induce cytosolic Ca^2+^ exhaustion, which was quite different from TG. Combined with these data, we supposed that SBF-1 might inhibit the activity of SERCA and Ca^2+^ pumps in the plasma membrane (such as PMCA1), resulting in Ca^2+^ release from ER and accumulation in the cytoplasm, which might explain the strong antigrowth effects of SBF-1.

It has been reported that impairment of SERCA2 levels increase the sensitivity of cells to ER stress.^19, 34, 38, 39^ Coincidentally, knockdown of SERCA2 increased the sensitivity of HeLa cells to SBF-1, whereas overexpression of SERCA2 decreased the sensitivity of HeLa cells to SBF-1 (Figure 4 and Supplementary Figure S7). Furthermore, *in vivo* experiments indicated that a very low dose of SBF-1 (5 *μ*g/kg) markedly inhibited the growth of HeLa xenografts, with repression of SERCA activity and induction of abundant apoptosis and severe ER stress (Figure 5 and Supplementary Figure S9).

Aberrant SERCA expression has been reported to be relevant with the susceptibility and progression of colon cancer, lung cancer and liposarcoma.^13, 14, 15, 16, 17, 18, 19, 20, 21^ However, the correlation of SERCA2 with the malignance of cervical cancer is not reported before. The following experiments indicated that elevated SERCA2 expression was detected in malignant cervical carcinomas and lymph metastasis, with a positive correlation with the clinical stages of malignant cervical carcinomas (Figures 6a–c).

In all, as illustrated in Figure 6d, when cells are exposed to SBF-1, SERCA activity is suppressed, inducing depletion of ER Ca^2+^ and increase of cytosolic Ca^2+^, which disturbs ER folding capacity and increases unfolded and misfolded proteins, activating the signaling pathways of UPR response and causing ER stress-associated cell death. This study suggests that SERCA2 could be a therapeutic target in human cervical cancer and SBF-1 might be a novel SERCA inhibitor to induce cell death.

## Materials and Methods

### Materials

SBF-1 is a synthetic steroidal glycoside.^30^ For *in vitro* experiments, SBF-1 was dissolved in DMSO to a concentration of 20 mM (stock solution), and biotin-SBF-1 was dissolved in DMSO to a concentration of 10 mM (stock solution); for *in vivo* assay, SBF-1 was dissolved in DMSO to a concentration of 1 mg/ml (stock solution), and stored at −20 °C. Anti-phospho-eIF2*α* (no. 3597), anti-eIF2*α* (no. 9722), anti-CHOP (no. 5554) and anti-SERCA2 (no. 9580) antibodies were purchased from Cell Signaling Technology (Beverly, MA, USA). Anti-ATF6*α* (sc-22799), anti-PCNA (sc-56), anti-Ki-67 (sc-15402), anti-GAPDH (sc-166545), anti-*β*-actin (sc-47778) antibodies, goat anti-mouse IgG, goat anti-rabbit IgG and donkey anti-goat IgG HRP- conjugated antibodies and mouse, rabbit and goat IgG were purchased from Santa Cruz Biotechnology (Santa Cruz, CA, USA). Anti-XBP1 (no. 3166-1) and anti-*α*-tubulin (no. 1878-1) antibodies were purchased from Epitomics – an Abcam Company (Burlingame, CA, USA). Tissue arrays of human cervical cancer (CR2088, CR805 and CR806) were purchased from US Biomax Inc. (Rockville, MD, USA). Fibronectin (F2006) and PBA were purchased from Sigma Chemical Co. (St. Louis, MO, USA). Fluo-4 AM (F14217), BAPTA-am (B1205), Alexa Fluor 488 donkey anti-goat IgG (H+L) (A11055) and Alexa Fluor 594 goat anti-mouse IgG (H+L) (A11005) were purchased from Life Technologies (Carlsbad, CA, USA). 3-(4, 5-Dimethylthiazol-2-yl)-2,5-diphenyltetrazolium bromide (MTT) and DMSO were purchased from Sunshine Biotechnology (Nanjing, China). All other chemicals were purchased from Sigma Chemical Co.

### Cell culture

Human cervical cancer cell line HeLa, human umbilical vein endothelial cell line ECV304 and human T-lymphocyte cell line Jurkat were purchased from the Shanghai Institute of Cell Biology (Shanghai, China), respectively, maintained in Dulbecco's modified Eagle's medium (DMEM; Gibco, Grand Island, NY, USA) or in RPMI-1640 medium (Gibco) supplemented with 10% fetal bovine serum (FBS; Gibco), 100 U/ml penicillin and 100 mg/ml streptomycin, and incubated at 37 °C in a humidified atmosphere containing 5% CO_2_ in the air. Mouse T lymphocytes were separated from peripheral lymph nodes of C57BL/6 mice and maintained in RPMI-1640 medium (Gibco) supplemented with 10% FBS.

### Animals

Female C57BL/6 mice and female nude mice (6–8 weeks old) were obtained from the Shanghai Laboratory Animal Center (Shanghai, China). Briefly, mice were fed with free access to pellet food and water in plastic cages at 21±2 °C and kept on a 12-h light–dark cycle. Animal welfare and experimental procedures were carried out strictly in accordance with the Guide for the Care and Use of Laboratory Animals (The Ministry of Science and Technology of China, 2006) and the related ethical regulations of our university. All efforts were made to minimize animal suffering and to reduce the number of animals used.

### MTT assay

A total of 2 × 10^3^ cells were seeded into 96-well plates, and incubated with various concentrations of SBF-1 at 37 °C for indicated time periods. Four hours before measurement, 20 *μ*l per well MTT solutions (4 mg/ml in PBS) were added into the 96-well plates. After incubation at 37 °C for 4 h, the supernatants were discarded and 200 *μ*l per well DMSO was added. Then, the plates were measured under an FL × 800 Fluorescence Microplate Reader (BioTek, Winooski, VT, USA) at 570 nm. The optical density values of the wells without SBF-1 were considered as 100% cell growth rate.

### Migration and colony formation assay

A total of 2 × 10^5^ cells per well were seeded into 6-well plates and incubated with DMSO or various concentrations of SBF-1 for 48 h. For migration assay, the cells were washed once with serum-free DMEM and diluted to a concentration of 1 × 10^5^ per ml, and 0.1 ml cell suspension was added to the inner room of transwell (Costar) pretreated with 10 *μ*g/ml fibronectin. Next, 0.6 ml DMEM supplanted with 20% FBS was added into the wells of 24-well plates and the cells were incubated at 37 °C for another 24 h. Then, the cells were fixed with 4% paraformaldehyde (PFA) at room temperature for 30 min and stained with crystal violet (Sigma; 1% in distilled water) for 10 min. After washing two times with distilled water, migrated cells were counted with an optical microscope (Olympus, Shinjuku, Tokyo, Japan; five fields per transwell). For colony formation assay, 1 × 10^3^ cells per well were seeded into 6-well plates and incubated at 37 °C for another 2 weeks. Then, the cells were fixed with 4% paraformaldehyde at room temperature for 30 min and stained with crystal violet for 10 min (1% in distilled water). After washing two times with distilled water, photos were taken with a digital camera (Olympus), and the number of colonies, the cell number of which exceeded 80, were counted with an optical microscope (Olympus).

### Immunoblotting

In brief, cells were collected and washed with ice-cold PBS two times before being lysed in radio immunoprecipitation assay (RIPA) lysis buffer (Beyotime, Haimen, China; 50 mM Tris (pH 7.4), 150 mM NaCl, 1% Triton X-100, 1% sodium deoxycholate, 0.1% SDS, 1 mM phenylmethylsulfonylfluoride, 0.15 U/ml aprotinin and 1 mg/ml pepstatin). Whole-cell lysates were collected and proteins were resolved by SDS-PAGE and were then electrotransferred onto polyvinylidene fluoride membranes. Then, the membranes were blocked in 5% bovine serum albumin (Sigma) at room temperature for 1 h, and the blots were incubated with primary antibody at 4 °C overnight and with secondary antibody at room temperature for 1 h. After extensive washing, the blots were developed with a chemiluminescence assay system (Cell Signaling Technology) and exposed to films (Kodak, Rochester, NY, USA) for appropriate time periods. The densitometry of immunoblots was quantified with Image J software (NIH, Bethesda, MD, USA) and normalized with loading controls.

### Reverse transcription and quantitative real-time PCR

Total RNA was extracted from cells or animal tissues using Trizol reagent (Life Technologies) according to the manufacturer's instructions. The reverse transcription with 1 *μ*g of total RNA was conducted under the following conditions: 42 °C for 20 min and 99 °C for 5 min. The abundance of gene expression was then analyzed with SsoFast EvaGreen Supermix (Bio-Rad, Berkeley, CA, USA) on CFX96 Touch Real-Time PCR Detection System (Bio-Rad; 94 °C, 15 s; 58 °C, 30 s; 40 cycles) and normalized with 18S ribosome RNA (*RN18S*) level. The primer sequences used were as follows (5′–3′): *CALNEXIN* – sense, CCAAGGTTACTTACAAAGCTCCA and antisense, GGCCCGAGACATCAACACA; *CALRETICULIN* – sense, CCTGCCGTCTACTTCAAGGAG and antisense, GAACTTGCCGGAACTGAGAAC; *CHOP* – sense, GGAAACAGAGTGGTCATTCCC and antisense, CTGCTTGAGCCGTTCATTCTC; *GRP78* – sense, CATCACGCCGTCCTATGTCG and antisense, CGTCAAAGACCGTGTTCTCG; *GRP94* – sense, GCTGACGATGAAGTTGATGTGG and antisense – CATCCGTCCTTGATCCTTCTCTA; *GRP170* – sense, GAGGAGGCGAGTCTGTTGG and antisense, GCACTCCAGGTTTGACAATGG; *SERCA1* – sense, GTGATCCGCCAGCTAATG and antisense, CGAATGTCAGGTCCGTCT; *SERCA2a –* sense, CTGTCCATGTCACTCCACTTCC and antisense, TTACTCCAGTATTGCAGGT; *SERCA2b* – sense, ACCAAATCCTGCTCGTTC and antisense, ATCGCTAAAGTTAGTGTCTGTG; *SERCA3* – sense, GATGGAGTGAACGACGCA and antisense, CTCTTCTTCCGATACCTGG; *SERCA2* – sense, GGAACCCAAAGGAACCAT and antisense, AACAGCCAATAGCCAAGT.

### Competitive binding assay

HeLa whole-cell lysates were, respectively, incubated with 10 *μ*M biotin, 10 *μ*M biotin-SBF-1, 10 *μ*M biotin-SBF-1 plus 100 *μ*M SBF-1 or 10 *μ*M biotin-SBF-1 plus 200 *μ*M SBF-1 and 50 *μ*l streptavidin-conjugated sepharose beads (GE Healthcare) at 4 °C overnight for 12 h. Then, the mixture was centrifuged at 4000 × *g* for 1 min to obtain the precipitation. After washing five times with RIPA lysis buffer, the beads were boiled in 2 × loading buffer (100 mM Tris-HCl (pH 6.8), 4% SDS, 1% bromphenol blue, 20% glycerol and 2% *β*-mercaptoethanol). After centrifugation at 4000 ×  *g* for 2 min, the supernatant were collected and separated with SDS-PAGE.

### Silver staining and LC/MS

SDS-PAGE gels were fixed (40% ethanol, 10% acetic acid in distilled water) at room temperature (RT) overnight. Then, the gels were washed once with distilled water and sensitized (150 ml ethanol, 34 g anhydrous sodium acetate, 1.57 g anhydrous sodium thiosulfate and metered to 500 ml with distilled water) RT for 30 min. After washing two times, the gels were silver-stained (0.125 g silver nitrate and metered to 50 ml) at RT for 20 min. Next, the gels were washed once with distilled water and colorized with formaldehyde (20 *μ*l in 50 ml distilled water) for appropriate time periods. Finally, the reaction was stopped by adding ethylene diamine tetraacetic acid (0.1% in distilled water). Interested bands were cut and analyzed with LC/MS, which was performed by Shanghai Institute of Biochemistry Proteomics Center (Shanghai, China).

### Immunofluorescence

Cells incubated with 0.1% DMSO or 10 *μ*M biotin-SBF-1 for 1 h were fixed in 4% paraformaldehyde (pH 7.4) for 30 min. Then, the cells were softly washed two times with PBS, and incubated with 0.5% Triton X-100 for 30 min. Thereafter, cells were softly washed two times with PBS and blocked with 3% bovine serum albumin for 1 h. Cells were incubated with primary antibodies (anti-SERCA2 and anti-biotin antibodies, respectively) at 4 °C overnight. After washing two times, the cells were incubated with secondary antibodies for 1 h and the nucleus was stained with 4, 6-diamidino-2-phenylindole (DAPI) for 1 min. The fluorescent signals were detected with a mercury lamp (Olympus U-RFL-T) and analyzed by Image Pro Plus 6.0 software (Media Cybernetics Inc., Rockville, MD, USA).

### Detection of cytosolic Ca^2+^ signals

Cells pretreated with DMSO or various concentrations of SBF-1 for 48 h were collected and stained with Fluo-4 AM (2.5 *μ*M in Hank's balanced salt solution (HBSS); Gibco) for 60 min. Then, the cells were washed two times with HBSS and cytosolic Ca^2+^ signals were detected with FACS Calibur flow cytometry (FACS; BD Biosciences, San Jose, CA, USA) or a mercury lamp (Olympus U-RFL-T).

### Measurement of Ca^2+^-ATPase (SERCA) activity

Cells were incubated with DMSO or various concentrations of SBF-1 for 48 h. Then, the cells were washed once with cold saline (0.9% sodium chloride in distilled water) and resuspended with saline. After sonicated for 10 s, SERCA activity of the suspension was measured with Super Microscale Ca^2+^-ATPase Detection Kit (A070-4; Nanjing Jiancheng Bioengineering Institute, Nanjing, China) according to the manufacturer's protocols.

### Plasmids and lentivirus

Construction of pcDNA3.1(+)-hSERCA2b was performed as before.^19^ Construction of sh-SERCA2 lentivirus was performed by Neuron Biotechnology (Shanghai, China). The vector of the shRNA was pLKD-CMV-G&PR-U6 and the map was shown in Supplementary Figure S1; the sequence was 5′-CAAAGUUCCUGCUGAUAUA-3′. Control lentivirus particles were provided by Neuron Biotechnology and the sequence was 5′-UUCUCCGAACGUGUCACGU-3′. For transient transfection, cells were seeded into six-well plates and allowed to grow to 40% confluence. Then, 2 *μ*g pcDNA3.1(+) or pcDNA3.1(+)-hSERCA2b together with 2 *μ*l Lipofectamine 2000 (Life Technologies) were added into the cells, respectively. Twenty-four hours after transfection, the cells were incubated with DMSO or various concentrations of SBF-1 for another 48 h. For lentivirus infection, 1 × 10^5^ cells in a well were seed into 12-well plates. Twelve hours later, 1 × 10^6^ plaque-forming units of control lentivirus particles or sh-SERCA2 lentivirus particles were added to the cells, respectively. Three days after infection, the infected cells were incubated with 8 *μ*g/ml puromycin (P8833; Sigma) for another 2 weeks. Then, the cells were harvested and the protein levels of SERCA2 were detected with immunoblotting. Cells stably infected with control or sh-SERCA2 lentivirus were incubated with DMSO at various concentrations of SBF-1 for 48 h. Then, the cells were collected for the following experiments.

### *In vivo* tumor xenografts model

One million HeLa cells (in 0.1 ml PBS) were subcutaneously injected into the right flanks of nude mice. Seven days later, all the mice formed visible tumors. Then, the mice were distributed into two groups according to tumor volumes. Vehicle (0.1% DMSO in PBS, *n*=5) and 5 *μ*g/kg SBF-1 (*n*=6) were daily intraperitoneally injected to the tumor-bearing mice for 12 days. Body weight and tumor volumes were measured and recorded every 2 days. Long diameter (*L*) and short diameter (*S*) of a tumor were measured with a vernier caliper and the tumor volume was calculated as follows: *L* × *S*^2^/2. Twelve days after injection, the mice were killed and tumors were separated. Tumor weight was measured and tumor sections were fixed in formalin. The rest of the sections were frozen in liquid nitrogen and stored at −80 °C.

### Immunohistochemistry and TUNEL staining

Paraffin sections of xenografts were cut into 5- *μ*m-thick sections. Then, the sections were infused in xylene (10 min per time for three times), followed by infusion in 90% ethanol (3 min), 75% ethanol (3 min), 50% ethanol (3 min) and ddH_2_O (3 min). For immunohistochemistry, the sections were boiled in antigen restoration solution (P0081; Beyotime) for 3 min and cooled naturally. Cooled sections were incubated with 3% H_2_O_2_ at RT for 10 min and blotted with 3% horse serum for 1 h. Next, the sections were incubated with primary antibodies at 4 °C overnight. The following steps were in accordance to the protocols provided by the manufacturer of GTVision III Detection System/Mo&Rb (GK500705; Gene Tech Company Limited, Shanghai, China). The nucleus was stained with hematoxylin. TUNEL staining was performed according to the instructions provided by the manufacturer of DeadEnd Fluorometric TUNEL System (TB235; Promega, Madison, WI, USA). The nucleus was stained with DAPI (Beyotime) for 1 min and the percentage of TUNEL-positive cells was calculated with a mercury lamp (Olympus U-RFL-T).

### Statistical analysis

Data are expressed as means±S.D. One-way ANOVA was used to evaluate the differences among more than three groups. The Student's *t*-test was used to evaluate the difference between two groups. *P*<0.05 was considered to be significant.

## Abbreviations

SERCA2: sarco/endoplasmic reticulum Ca2+-ATPase 2
ER: endoplasmic reticulum
BAPTA: 1,2-bis(o-aminophenoxy)ethane-*N*,*N*,*N*′,*N*′-tetraacetic acid
PBA: 4-phenyl-butyric acid
CHOP: C/EBP homologous protein
eIF: eukaryotic translation initiation factor
GRP: glucose-regulated protein
ATF: activating transcription factor
XBP: X-box binding protein
MTT: 3-(4,5-Dimethylthiazol-2-yl)-2,5-diphenyltetrazolium bromide
DMSO: dimethyl sulfoxide
