# Supplementary material for: SBF-1 exerts strong anticervical cancer effect through inducing endoplasmic reticulum stress-associated cell death via targeting sarco/endoplasmic reticulum Ca2+-ATPase 2
Source: Cell Death Dis. 2014 Dec 18;5(12):e1581–. doi: 10.1038/cddis.2014.538 (PMC4649847; doi:10.1038/cddis.2014.538)
Supplement: Supplementary Information [file cddis2014538x1.doc]

**SBF-1 exerts strong anti-cervical cancer effect through inducing endoplasmic reticulum stress-associated cell death via targeting sarco/endoplasmic reticulum Ca2+-ATPase 2**

Wanshuai Li^1, 4^, Zijun Ouyang^1, 4^, Qi Zhang^1^, Lu Wang^1^, Yan Shen^1^, Xudong Wu^1^, Yanhong Gu^2^, Yongqian Shu^2^, Biao Yu^3^, Xuefeng Wu^1,*^, Yang Sun^1,*^ and Qiang Xu^1,*^

^1^State Key Laboratory of Pharmaceutical Biotechnology, School of Life Sciences, Nanjing University, 22 Hankou Road, Nanjing 210093, China

^2^Department of Oncology, The First Affiliated Hospital of Nanjing Medical University, Nanjing 210029, China

^3^State Key Laboratory of Bio-organic and Natural Products Chemistry, Shanghai Institute of Organic Chemistry, Chinese Academy of Sciences, Shanghai 200032, China

^4^ These authors contributed equally to this work.

**Supplementray materials**

*Correspondence to

Qiang Xu, PhD, or Yang Sun, PhD, or Xuefeng Wu, PhD, School of Life Sciences, Nanjing University, Nanjing 210093 China, Tel/fax: +86-25-83597620; e-mail: [molpharm@163.com](mailto:molpharm@163.com) (Q Xu); yangsun@nju.edu.cn (Y Sun); wuxf@nju.edu.cn (X Wu).

**
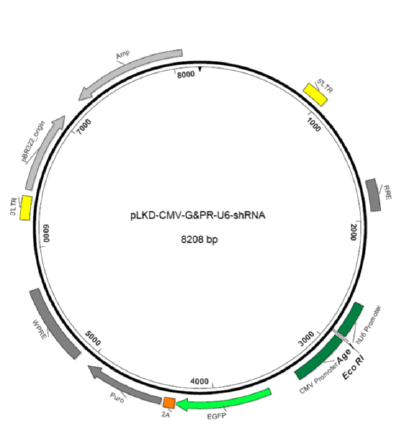
**

**Supplementary figure S1 Map of the shRNA vector provided by Nuro Biotech, Inc.**


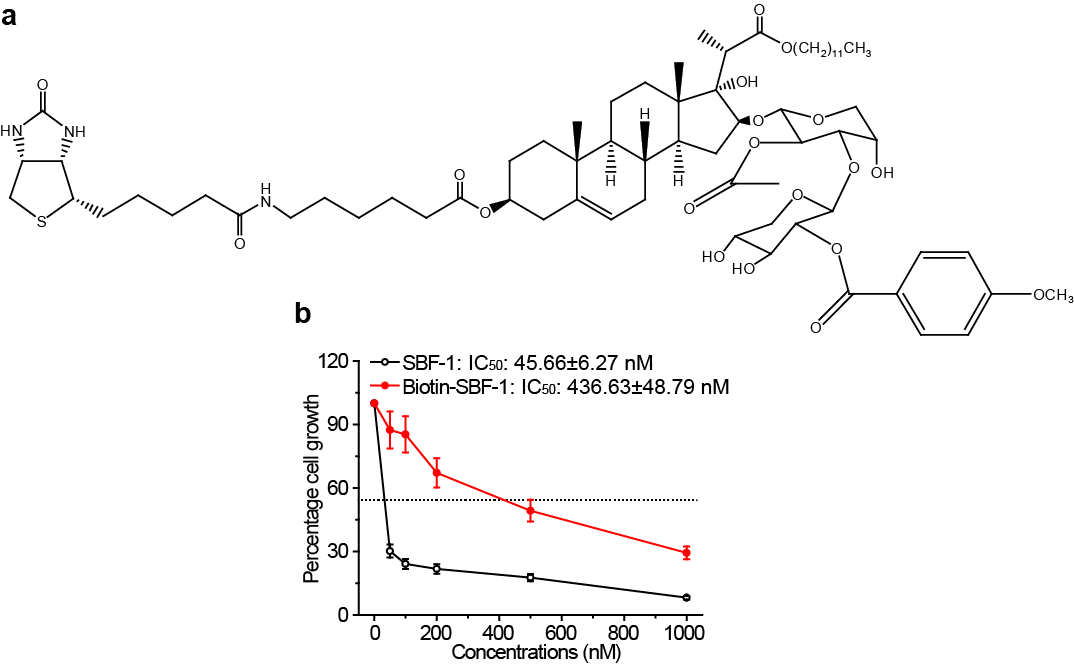


**Supplementary figure S2 Effects of SBF-1 and biotin-SBF-1 on the proliferation of Hela cells.** (a) Chemical structure of biotin-SBF-1. (b) Hela cells were seeded into 96-well plates and incubated with DMSO (0.1%) or various concentrations of SBF-1 or biotin-SBF-1 for 48 hours. Cell growth was determined by MTT assay. Data were means ± SD of three independent experiments. Dotted line indicated 50% cell growth of 0 nM SBF-1.


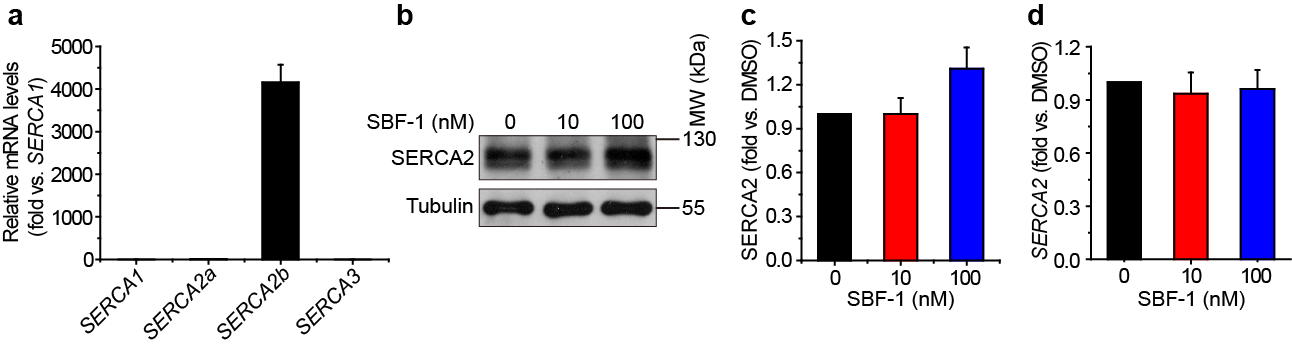


**Supplementary figure S3 Expression of SERCA isoforms and effects of SBF-1 on the protein and mRNA level of SERCA2 in Hela cells.** (a) The mRNA levels of SERCA1, SERCA2a, SERCA2b and SERCA3 were detected with Q-PCR and normalized with RN18S. (b-d) Hela cells were incubated with DMSO (0.1%) or various concentrations of SBF-1 for 48 hours. The protein levels of SERCA2 (b) was detected with immunoblotting and relative band intensity was analyzed by Image J software (NIH) and normalized with α-Tubulin (c). Blots shown were representative of three independent experiments. α-Tubulin was performed as a loading control. (d) The mRNA level of *SERCA2* was detected with Q-PCR and normalized with *RN18S*. Data were means ± SD of three independent experiments.


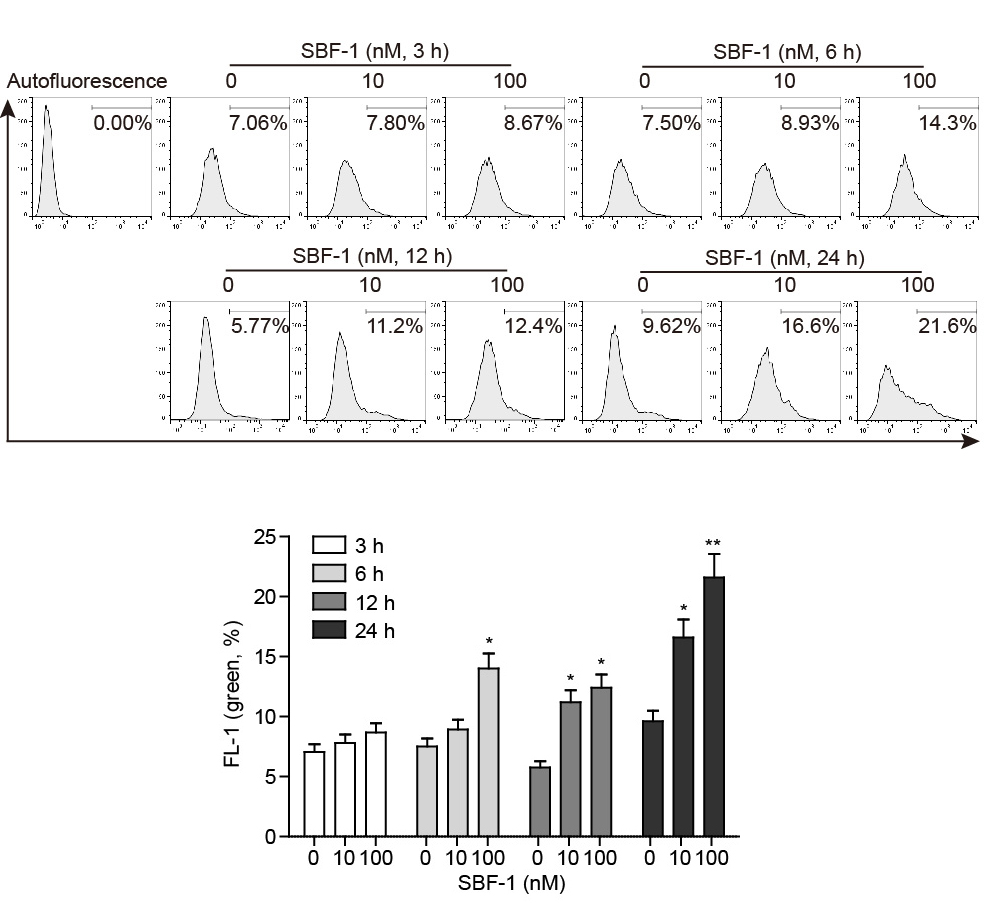


**Supplementary figure S4 SBF-1 increased cytosolic Ca2+ levels both in a concentration- and time-dependent manner.** Hela cells pretreated with DMSO (0.1%) or various concentrations of SBF-1 for various time periods. Then the cells were stained with Fluo-4 AM (2.5 μM). Percentage of Fluo-4 positive cells was analyzed with FACS. **P*<0.05, ***P*<0.01 vs. 0 nM SBF-1.


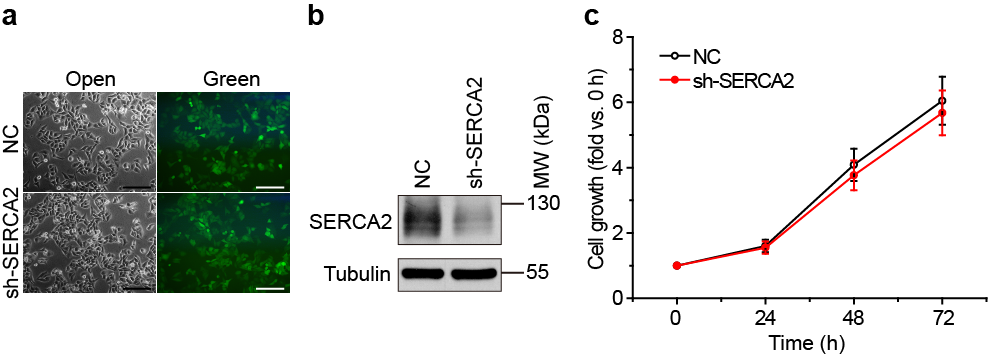


**Supplementary figure S5 Influences of SERCA2 knockdown on the proliferation of Hela cells.** (a) Microscopic images of Hela cells stably infected with NC shRNA or SERCA2 shRNA. Images were representative of three independent experiments. (b) Protein level of SERCA2 in Hela cells stably infected with NC shRNA or SERCA2 shRNA was detected with immunoblotting. Blots shown were representative of three independent experiments. (c) Hela cells stably infected with NC shRNA or SERCA2 shRNA were seeded into 96-well plates and maintained at 37 ^o^C for various time periods. Cell growth was determined by MTT assay. Data were means ± SD of three independent experiments.


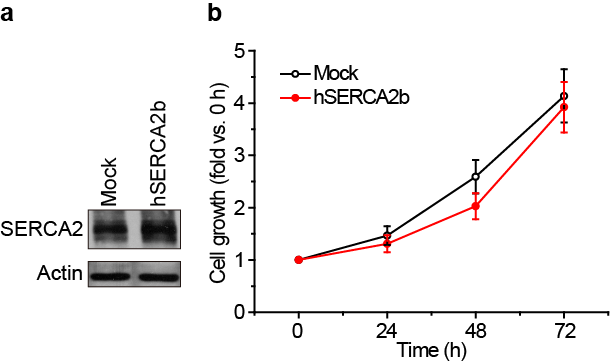


**Supplementary figure S6 Influences of SERCA2 overexpression on the proliferation of Hela cells.** Hela cells transiently transfected with mock (pcDNA3.1(+)) or hSERCA2b (pcDNA3.1(+)-hSERCA2b) for 48 hours were seeded into 96-well plates and maintained at 37 ^o^C for various time periods. (a) The protein level of SERCA2 was detected with immunoblotting and representative bands of three independent experiments were shown. (b) Cell growth was determined by MTT assay. Data were means ± SD of three independent experiments.


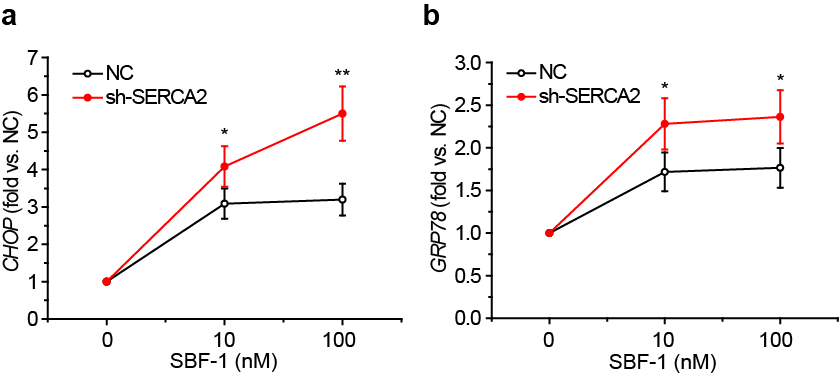


**Supplementary figure S7 Knockdown of SERCA2 increased SBF-1-induced ER stress in Hela cells**. Hela cells stably infected with NC shRNA and SERCA2 shRNA were incubated with DMSO (0.1%) or various concentrations of SBF-1 for 48 hours. (a and b) The mRNA levels of *CHOP* (a) and *GRP78* (b) were detected with Q-PCR and normalized with *RN18S*. Data were means ± SD of more than three independent experiments. * *P*<0.05, ** *P*<0.01 vs. NC.


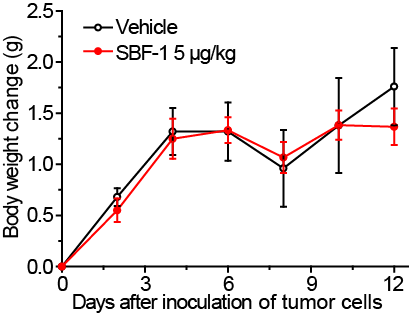


**Supplementary figure S8 Influences of SBF-1 on the body weight of tumor-bearing mice.** One million cells (in 0.1 ml PBS) were injected subcutaneously into the right flanks of female nude mice (6-8 weeks old). One week after the inoculation, all the mice formed visible tumors and were distributed into two groups. Vehicle (0.1% DMSO in PBS, n=5) and 5 μg/kg SBF-1 (n=6) were intraperitoneally injected into the mice. Body weight of tumor-bearing mice was measured every two days. Data were means ± SD of five to six mice.


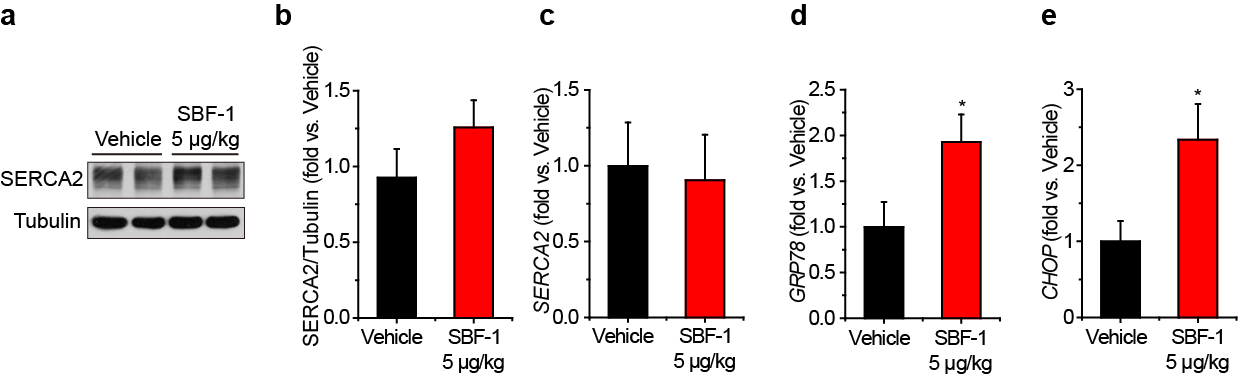


**Supplementary figure S9 Effects of SBF-1 on the protein and mRNA levels of SERCA2.** One million cells (in 0.1 ml PBS) were injected subcutaneously into the right flanks of female nude mice (6-8 weeks old). One week after the inoculation, all the mice formed visible tumors and were distributed into two groups. Vehicle (0.1% DMSO in PBS, n=5) and 5 μg/kg SBF-1 (n=6) were intraperitoneally injected into the mice. (a and b) The protein level of SERCA2 was detected with immunoblotting (a), and relative band intensity was analyzed with Image J software (NIH) and normalized with α-Tubulin (b). (c-e) The mRNA levels of *SERCA2*, *GRP78* and *CHOP* were detected with Q-PCR and normalized with *RN18S*. Data were means ± SD of five to six mice. * *P*<0.05 vs. Vehicle.


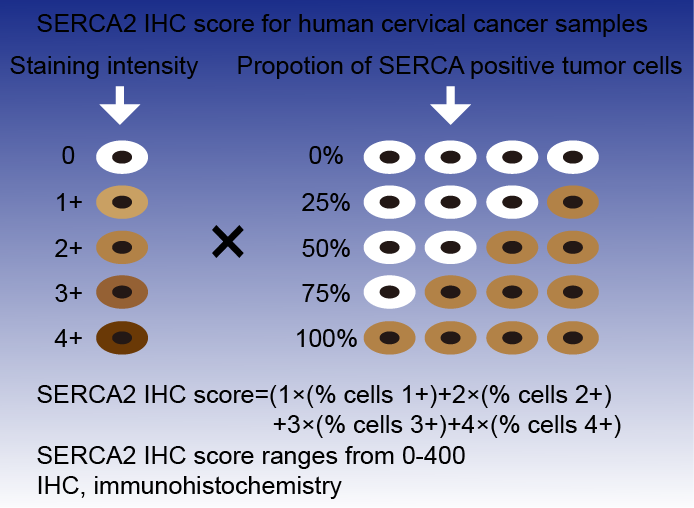


**Supplementary figure S10 Evaluation of SERCA2 IHC score as described before with minor modifications.**[**^1^**](#_ENREF_1)


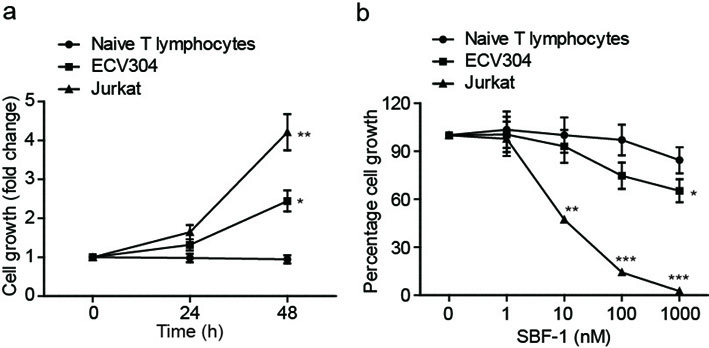


**Supplementary figure S11 The anti-growth effects of SBF-1 on slowly dividing cells (naïve T cells and ECV304 cells) and rapidly dividing cancer cells (Jurkat cells).** Naïve T cells, ECV304 cells and Jurkat cells (2×10^3^/well) were seeded into 96-well plates and incubated with medium (a) or various concentrations of SBF-1 (b) for 48 hours. Then cell growth was determined by MTT assay. Data were means ± SD of three independent experiments. **P*<0.05, ***P*<0.01 vs. medium (a) or 0 nM SBF-1 (b).

**References**

1. Hashimoto K, Saigusa S, Araki T, Tanaka K, Okita Y, Fujikawa H*, et al.* Correlation of CCL20 expression in rectal mucosa with the development of ulcerative colitis-associated neoplasia. *Oncology letters* 2013, **6**(5)**:** 1271-1276.
